# A system for determining Li-ion cell cooling coefficients

**DOI:** 10.1016/j.ohx.2022.e00257

**Published:** 2022-01-07

**Authors:** Felix Russell, Alastair Hales, Gavin White, Yatish Patel

**Affiliations:** a Electrochemical Science and Engineering Research Group, Mechanical Engineering Department, Imperial College London, London, SW7 2BX, UK; b Department of Mechanical Engineering, University of Bristol, Bristol, UK

**Keywords:** CCC, Cell cooling coefficient, Peltiers, Peltier elements, BOM, Bill of materials, OSF, Open science framework, XPS, Expanded polystyrene, Electrochemistry, Battery thermal management, Power systems, Novel test methods

## Abstract

Current battery data sheets focus on battery energy and power density, neglecting thermal performance. This leads to reduced system level efficiency since cells with poor thermal performance require larger, heavier cooling systems to maintain cell temperatures in a suitable range. To address this a new metric, the Cell Cooling Coefficient (CCC), has been developed and it’s use as a tool for appropriate cell selection has been demonstrated. It also allows the pack designer to calculate which cooling direction method is most suitable by comparing CCC values for tab and surface cooling.

The metric is the ratio between the heat rejected from the cell and the temperature difference between the hottest and coolest point. It therefore has units $W\;K^{-1}$ and allows a pack designer to easily calculate the required amount of cooling power for the cell given a maximum acceptable temperature rise. In this paper we describe a system and method for the accurate determination of the CCC with the aim of facilitating wider adoption of the metric. The system is able to reliably quantify the surface and tab cooling CCC of any pouch cell.

## Graphical abstract

**Figure:**
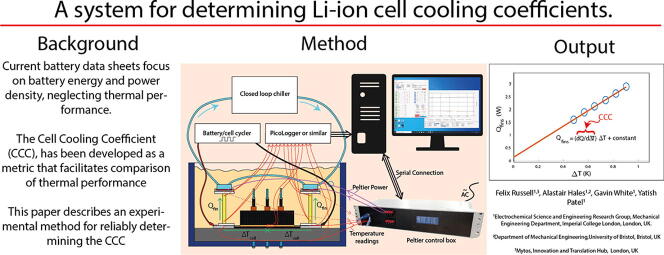

**Specifications table:**

**Table:** 

**Hardware name**	Pouch Cell Cooling Coefficient (CCC) measurement device

**Subject area**	• Engineering • Power systems • Battery thermal management • Electrochemical engineering
**Hardware type**	• Measuring physical properties
**Open source license**	*Creative Commons Attribution-ShareAlike license*
**Cost of hardware**	£335 up front + approximately £15 per cell tested. Capital cost of required commercially available lab equipment not included.
**Source file repository**	DOI 10.17605/OSF.IO/ZYWVF

## Hardware in context

Lithium-ion cells generate heat during use that must be removed to avoid temperature build up [1]. Unacceptably high cell temperatures degrade the cell, reducing battery life times, and, in extremis, can lead to dangerous thermal runaway [2]. Thermal control is becoming increasingly critical as cells are used in high power applications such as electric vehicles, where high peak currents generate greater amounts of heat. For many applications, cells represent a large proportion of the cost and embedded carbon footprint and are a key component that must be managed safely [3]. Therefore, increasing cell life and operational safety has implications on viability in the real-world. Overcoming existing limitations is essential for society to transition to these technologies [4].

Despite the implications of poor thermal management, cell data sheets do not provide information on battery thermal performance. Instead, they focus on energy and power per unit mass or volume. Power system designers have to infer thermal performance from cell dimensions and estimates of thermal properties for each material in the cell. A new metric, the Cell Cooling Coefficient (CCC) provides a single measure of thermal performance which can be used to compare the thermal performance of any cell against another [5], [6]. Specifically, it gives the required heat removal rate for a maximum acceptable temperature gradient across the cell. Two thermal management methods are commonly used for cells: cooling through the positive and negative electrodes, termed ‘tab cooling’; and cooling through the cell surface, termed ‘surface cooling’. These will produce different CCC values for a given cell, and comparison of the two will aid selection of the most appropriate method for the cell [7].

The basis for the system is the measurement of heat flow away from the measured surface (cell tabs or cell surface) under steady state conditions while the cell is generating heat from through an alternating current drive cycle. To truly mimic a particular thermal management method, all other surfaces are insulated throughout the test. The heat flows are measured by recording the temperature gradient along brass fins of known thermal resistance. Peltier elements (Peltiers) are used to maintain constant temperature boundary conditions, and the test lasts for long enough to ensure thermalisation throughout the apparatus. At this ‘elevated steady state’, the assumption that cell heat generation is equal to cell heat rejection is valid. The test method has been discussed and evaluated in three papers [5], [6], [7]. However, previous methods required the design of cell-specific parts (insulation, tab clamps and cooling fins) that fit the cell in question. Conversely, the hardware described in this paper facilitates measurement of CCC values for any pouch cell. Only a cell-specific sheet of aluminium (for the surface cooling CCC) and cell-specific insulating foam (for the tab cooling CCC) are required.

## Hardware description

The CCC is calculated by comparing the sum of heat flow through all the fins, $\Sigma Q_{\mathrm{fin}}$, with the temperature gradient across the cell, $\Delta T_{\mathrm{cell}}$. For accurate measurement of CCC it is critical that all other surfaces are well insulated such that heat loss through the fins is the dominant heat rejection pathway.

### Apparatus assembly

**Fig. 1:**
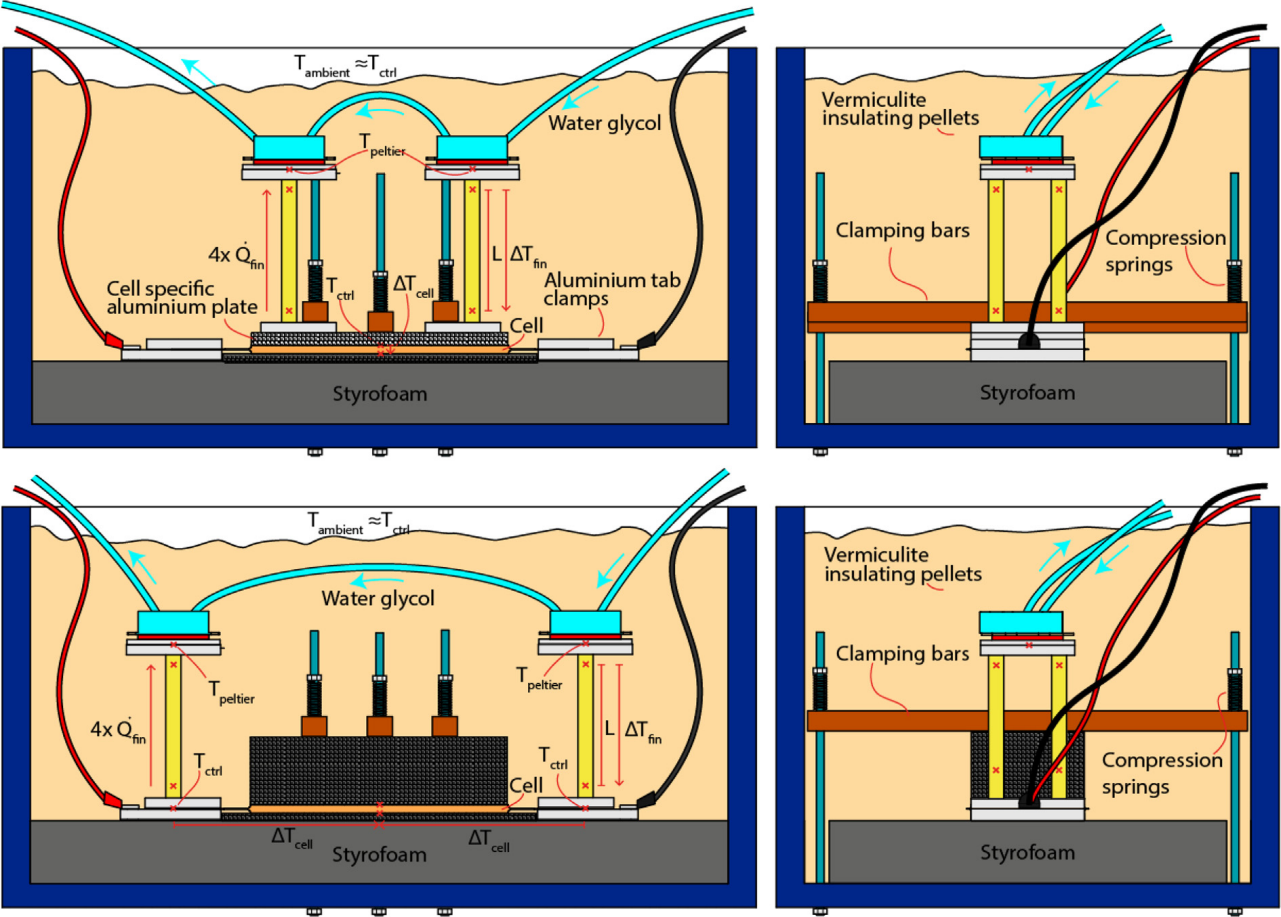
Cell assembly shown in the (A) tab cooling and (B) surface cooling configuration. Hashed parts are cell-specific.

Fig. 1 shows the cell in it’s insulated box. In the tab cooled case the fins are clamped to the positive and negative tabs and the upper surface of the cell is insulated. In the surface cooled case an aluminium plate covers the upper surface of the cell on top of which are the fins. In both cases clamping bars apply even pressure to the cell surface. During testing the box is filled with vermiculite ($0.06\;W\;m^{-1}\;K^{-1}$ [8]) to insulate the fins. Critical to this design is the ability to reuse the hardware for a large range of pouch cell sizes. To achieve this the clamping bar locations can be selected from any of 17 positions evenly spaced over the length of the box. The fins are designed to clamp onto any tab with a width and length smaller than 80mm. Peltier elements at the distal end of the fins remove heat from the conductive system. The heat energy is carried away via the water cooled blocks.

The designs for two sets of fins are published alongside this paper (CCC–CAD.zip). ‘Fin set 1’ for measuring 2–4 W of heat, ‘Fin set 2’ for measuring 1–2 W heat. In most tests, two of these fin sets are used (i.e. one set on each tab or alternatively two sets sitting on the cell surface), giving a cell heat production range of 2–8 W with the equipment specified. Whilst difficult to specify an exact range, due to the unique composition and thus heat generation characteristics or a specific cell, our preliminary investigations have found that this range is practical for cells with a nominal capacity between 5Ah and 80Ah. This covers almost all pouch cells that are currently commercially available. It would however be simple to specify fins for higher or lower heat rates by following the methods described in Section 5.

### CCC calculation

The heat passing through a single fin is described by Eq. 1 where $Q_{\mathrm{fin}}$ (Fig. 1) is the heat passing through the fin, $\Delta T_{\mathrm{fin}}$ is the temperature gradient along a fin, ΔL is the distance between temperature measurements, *A* is the cross-sectional area of the fin and *k* is the thermal conductivity of the fin material.

(1) $$Q_{\mathrm{fin}}=\frac{\Delta T_{\mathrm{fin}}}{\Delta L}\mathrm{Ak}$$

**Fig. 2:**
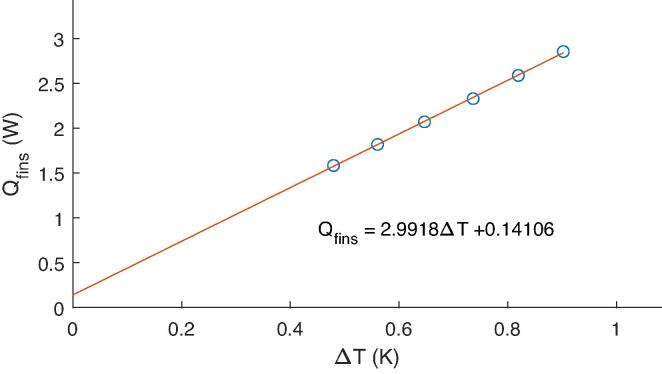
Kokam 7.5Ah cell test data under surface cooling. The ${\mathrm{CCC}}_{\mathrm{surf}}$ here is 2.99W/K.

The CCC is calculated from the total heat loss through all fins, $\Sigma Q_{\mathrm{fin}}$, and the temperature gradient across the cell, $\Delta T_{\mathrm{cell}}$, once thermalisation has been achieved in a test. The test is repeated with multiple magnitudes of current using an alternating current drive cycle, such that a graph of $\Sigma Q_{\mathrm{fin}}$ against $\Delta T_{\mathrm{cell}}$ can be plotted (Fig. 2). The CCC is then the gradient of a line of best fit through these points. In the case of tab cooling, $\Delta T_{\mathrm{cell}}$ is the difference between the temperature of the cell surface at the point furthest from the tabs and the average of the two tab temperatures. For surface cooling, $\Delta T_{\mathrm{cell}}$ is the difference in temperature between the top and bottom surfaces of the cell.

### Additional lab equipment required

**Fig. 3:**
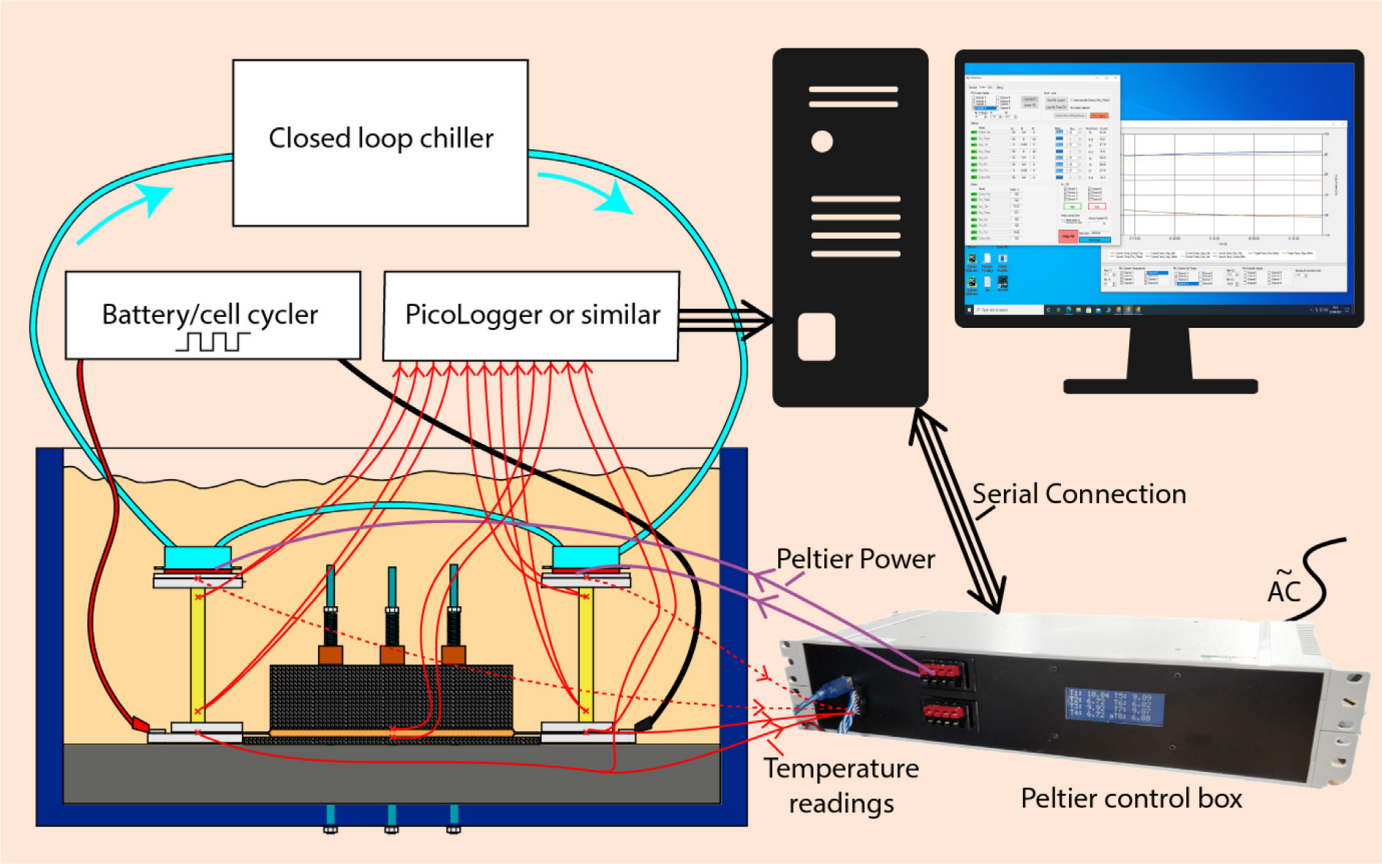
Schematic of the control system for cell temperature. For most Peltier control systems the measurement shown with the dotted line is not required.

A schematic of all elements is given in Fig. 3. To perform measurement of CCC the experimentalist must be equipped with:

• A galvanostatic-ready battery cycler with sufficient current magnitude capability to generate the required amount of heat during the alternating current drive cycle (approximately 2C for energy-dense cells and approximately 4C for power-dense cells). The alternating current drive cycle must have a frequency of at least 0.1 Hz (i.e. switching from charge to discharge or vice versa every five seconds) so that the cell’s state of charge can be taken as a constant throughout the test.
• A voltmeter capable of reading at the μV level for calculating the contact resistance between the wires and cell tabs.
• A closed loop chiller with cooling power exceeding the maximum heating power of the two Peltier elements.
• A device for recording temperature measurements from thermocouples of thermistors (e.g. a *Pico Technology TC-08* data logger).
• A Peltier controller. These systems are available commercially and control the current applied to a Peltier element in response to feedback from a temperature sensor in order to maintain constant temperature conditions. Measurement of CCC must occur under steady state thermal conditions, where cell heat generation is equal to cell heat rejection, and thus there is no heat gain or loss for the thermal masses of the insulation, cell or fins. The temperature sensor should be placed on the tabs for tab cooling or the top surface of the cell for surface cooling. The particular controller shown here uses additional feedback from under the Peltier elements to reduce temperature oscillations caused by changes in the external temperature [9]. Repeats of the validation were conducted without this feedback and produced comparable results when the lab temperature remained constant.

### Summary

• The Cell Cooling Coefficient (CCC) has been shown to provide information critical for the design of battery pack thermal management systems.
• The apparatus described in this paper provides a means to accurately measure CCC for the majority of sizes of pouch cell.
• The device can be used to measure the CCC of the cell through it’s tabs or surface, providing a means to compare these two thermal management methods.

## Design files

### Design Files Summary

.

**Table:** 

**Design filename**	**File type**	**Open source license**	**Location of the file**

*CCC–CAD.zip*	*CAD files*	Creative Commons Attribution-ShareAlike	DOI 10.17605/OSF.IO/ZYWVF
*CCC-BOM.xlsx*	*CAD files*	Creative Commons Attribution-ShareAlike	DOI 10.17605/OSF.IO/ZYWVF

The Open Science Framweork (OSF) depository given above contains CAD and drawing files referred to in this document (CCC–CAD.zip) and the Bill of Materials (BOM) (CCC-BOM.xlsx).

## Bill of materials

The complete BOM is provided as a separate file (see above). Where it applies, the part numbers in the BOM are the same as those of the corresponding CAD file.

## Build instructions

### Fins

In the design, the selection of fin length and diameter has been chosen with the following factors taken into account:

• The temperature gradient along the fins should be 8K<ΔT<16K for a reasonable range of heat flow. This is a compromise between error induced by limitations in accuracy of temperature measurement requiring a large gradient and physical limitations from formation of condensation requiring that the cold end be $>5^{\circ }C$. N.B. heat gain through the insulation at the fin cold produces a fixed percentage error in the CCC which does not increase with decreasing cold end temperature.
• The location of the temperature measurement (estimated at ±0.5mm) requires that the thermistors be far enough apart to limit this error to 1%.

**Fig. 4:**
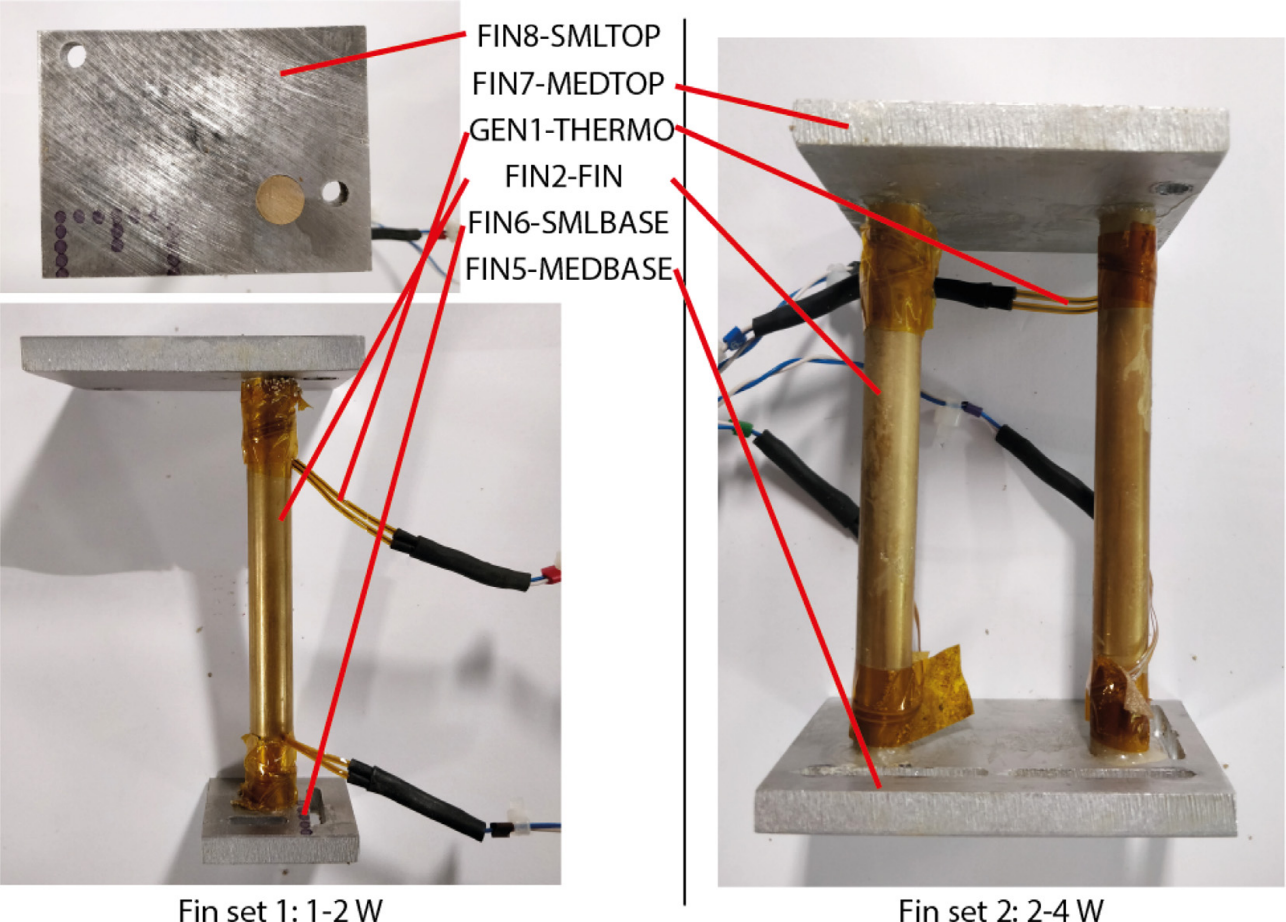
Construction of the fins. 1W-2W system shown on the left top down and side views). 2W-4W system shown on the right (side view only).

Eq. 1 can be used to determine the appropriate length and diameter dimensions. For large cells, which use two of the double sets (Fig. 4), the maximum heat generation was taken as $\overset{˙}{q}=8\;W$. By fixing L=100mm,ΔT<16K and $k=127\;W\;m^{-1}\;K^{-1}$ gives $A>406.5\;{\mathrm{mm}}^{2}$. This gives a diameter for each of the four fins of D>11.4mm. 12mm was chosen for this design but the same approach could be used to design different fins with other dimensions. The heat flow is likely to be inhomogeneous at the point at which it enters the fins. Our models show that a space of 5 mm from the aluminium plate to the thermistor is sufficient to ensure accurate heat flow measurement. To accommodate this and the 8mm plate thickness the brass bars must be cut to a length of 126mm.

1. The fins (Fig. 4) are constructed from brass round bar bonded to 8 mm aluminium plates with thermal epoxy (FIN1-EPOXY). The design of for the aluminium plates is given in design files FIN3-TABBTTM, FIN4-TABTOP, FIN5-MEDBASE, FIN6-SMLBASE, FIN7-MEDTOP and FIN8-SMLTOP. These can be milled or laser cut. For these, technical drawings in PDF format have been provided in addition to CAD files. If the plates are to be laser cut then the 12 mm holes should be cut undersize and drilled and then reamed to the correct diameter. N.B. FIN8-SMLTOP has both a left and right hand version. A fin set should be manufactured using each design.
2. Cut the fins (FIN3-FIN) to 126mm and removed any burs.
3. A pair of thermistors or thermocouples (GEN1-THERMO) should be attached 100 mm apart on each fin. Which you choose will depend on which measurement system you intend to use. The attachment should ideally be made with thermal epoxy (FIN1-EPOXY). Strain relief can be added by wrapping the cord round the fins and taping in place.
4. The fins (FIN3-FIN) should be glued (FIN1-EPOXY) into the aluminium plates so that they are all the way in the 12 mm holes flush with the far surface. For the medium sized double fin set the correct aluminium plates are FIN5-MEDBASE and FIN7-MEDTOP. For the small single fin set this is FIN6-SMLBASE and FIN8-SMLTOP. N.B. Check the correct plate orientation before gluing in file FinLayout.PDF.
5. Any burs remaining after machining should be filed off. The top and bottom surfaces of each fin set and all remaining aluminium plates should then be sanded flat with a sanding block and grit 240 sand paper.

### Insulating box

For this design a 600mm×400mm Euro container (INS1-BOX) encloses the cell and insulation. Other similarly sized boxes could be used instead.

1. Cut holes at intervals of 30mm to provide locations to attach clamping bars (Fig. 6). It may be useful to use technical drawing (INS1-BOX.PDF) as a stencil.

**Fig. 6:**
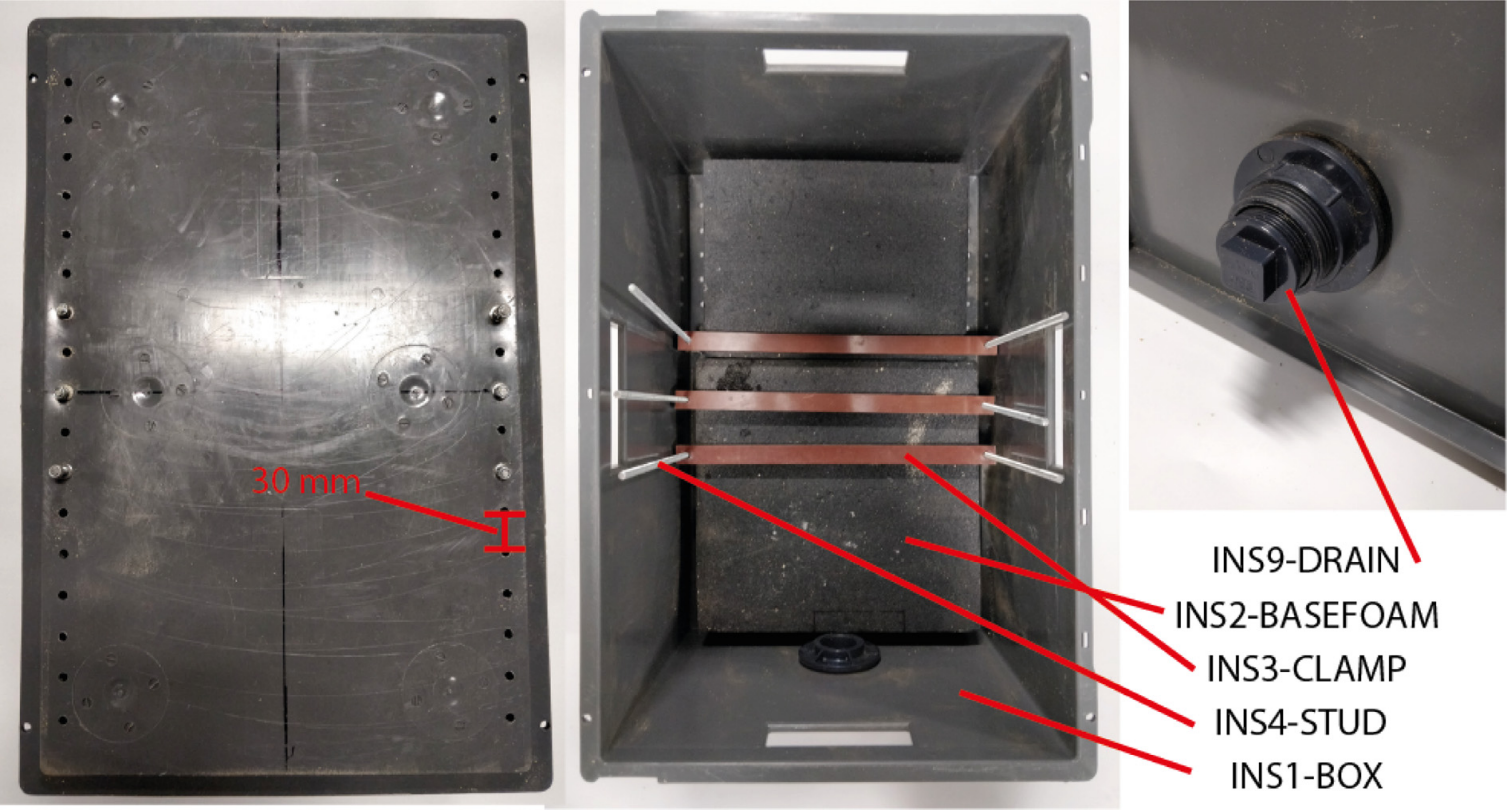
Construction of the insulating box.
2. A tank drain (INS9-DRAIN) is fitted to the box wall to facilitate removal of the vermiculite at the end of testing. Vermiculite does not readily flow so an inside diameter greater than 38mm is recommended. A hole saw should be used to make the appropriate cutout as near as possible to the bottom of the box at one end.
3. Place 75 mm thick grey expanded polystyrene (XPS) (INS2-BASEFOAM) foam ($k=0.03\;W\;m^{-1}\;K^{-1}$ [10]) in the bottom of the box to reduce heat losses. The foam should be narrow enough that is doesn’t cover the holes cut in step [1].
4. Clamping bars should be cut and drilled. The bar length will depend on the width of the chosen box. The part INS3-CLAMP is the correct size for the box specified under INS1-BOX in the BOM. For this design B12 Phenolic Cotton Laminate was chosen for it’s strength, toughness and electrical insulating properties (at low voltage). Any material with similar mechanical and electrical properties can be used here.
5. Cut the clamping rods (INS4-STUD) to the same height as the box (Fig. 5)

**Fig. 5:**
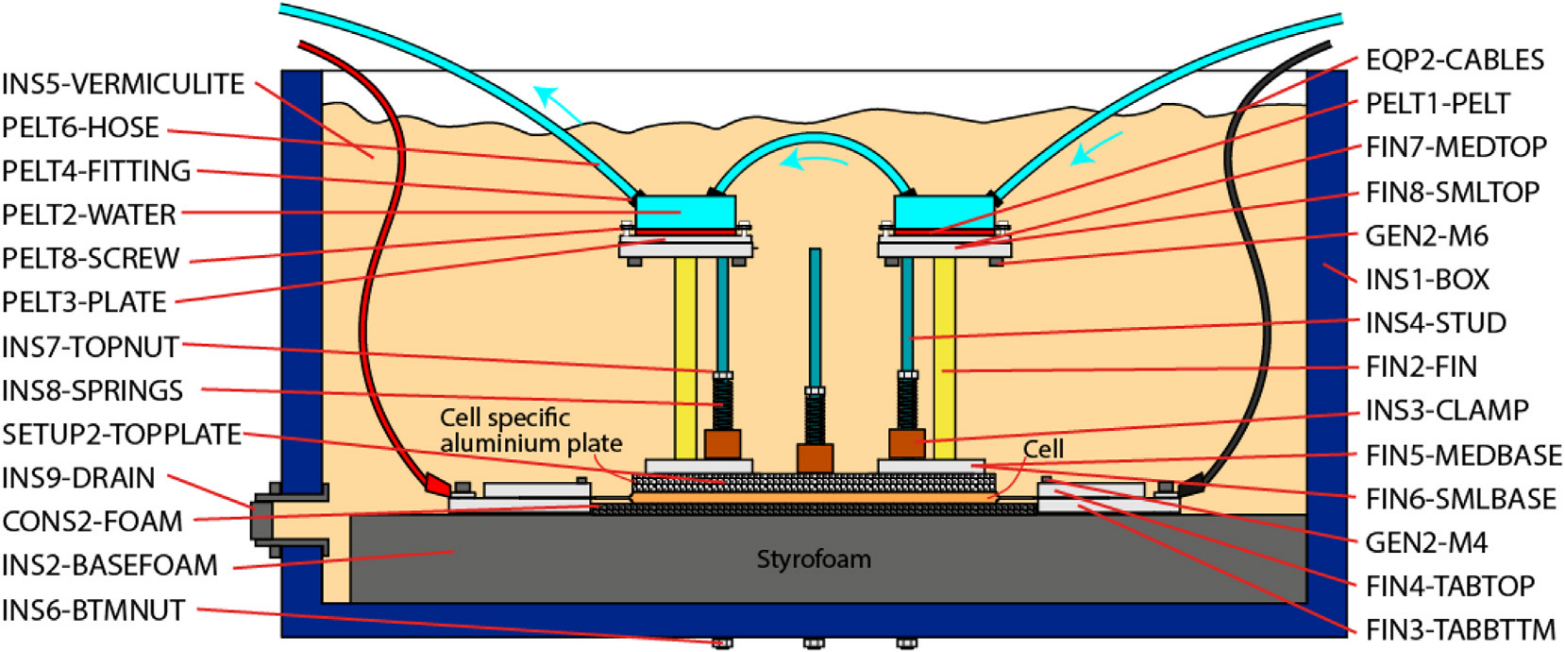
Schematic of the system in surface cooling mode. Labels corresponded to part numbers in the BOM and CAD file names (where applicable).

Pouch cells are designed to operate under pressure which is usually implemented during design of the battery pack. Compression springs (INS8-SPRINGS) with a rate of 3.61N/mm are used to apply the required force. The desired cell compression, unique for each cell and manufacturer, may be tuned by setting the length of the compressed spring to apply the correct force.

### Peltier assembly

1. Manufacture aluminium plate PELT3-PLATE. The plate holes should be tapped to M3 and M6 as per the technical drawing PELT3-PLATE.pdf. The M3 hole locations may need to be adapted if different water cooling blocks are used.
2. 40mm×40mm Peltier elements (PELT1-PELT) are placed on water cooling blocks (PELT2-WATER) with thermal interface material or paste at the interface (CONS1-INTERFACE).
3. Screw the Peltier and water cooling block to the aluminium plate with thermal interface material in between. Nylon screws (PELT8-SCREW) should be used here since their low thermal conductivity is critical for avoiding unwanted thermal short circuits across the Peltier element.

### Fin separator

**Fig. 8:**
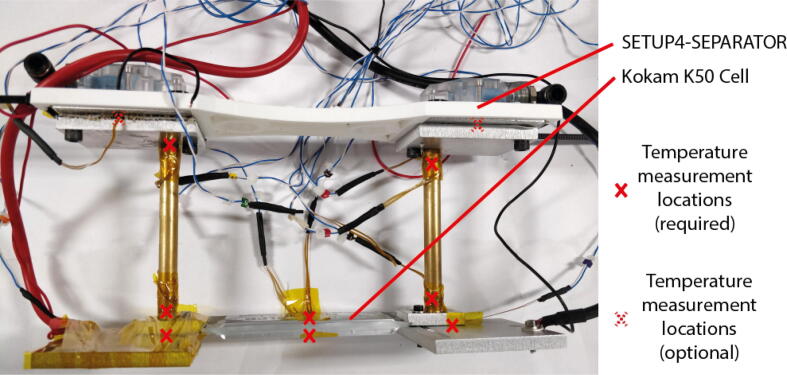
Kokam K50 with fin separator for tab cooling.

The fin separator (SETUP4-SEPARATOR) is necessary where cell tabs are close enough together that there is a risk that touching fins will cause a short circuit of the cell. For smaller cells this part may also make system assembly easier by combining both fin sets into a single rigid body. The part, which should be 3D printed, clips around the (PELT3-PLATE) under the water cooling blocks and is secured with a cable tie on each side (Fig. 8). Within the CAD file is a parameter “SEPARATION DISTANCE” that should be set to the desired separation of the Peltier plates. For fin set 2 and one orientation of fin set 1 the separation at the Peltier plates is the same as at the fin base.

## Operation instructions

Below are instructions for setup of the cell in the system and the procedure to begin testing. Low internal resistance Li-Ion cells can be dangerous if not handled correctly. Particular care should be taken to avoid short circuiting the tabs through the fins. In these instructions safety critical steps have been underlined however at all stages good judgement from users is required.

### Setup

The following describes the test setup procedure. It is recommended that as much of this setup at possible is performed outside the insulating box to facilitate good access to the cell surfaces.

1. Attach positive and negative battery cycler cables to the top surface of the tab bottom plates (FIN3-TABBTTM) making sure to clean the contacts with solvent (e.g. isopropanol).
2. Choose a pair of fin sets with size appropriate for the cell being tested. The large fin sets will be required where tabs are wider than 30mm or if the heat produced is likely to be greater than 4W for a >0.5K temperature gradient. This is often true for cells with capacity over 15 Ah.
3. Bolt a Peltier assembly to each fin set using the 6 mm clearance holes in the fin assembly top plate (FIN7-MEDTOP or FIN8-SMLTOP) and the M6 screw holes in the Peltier Plate (PELT3-PLATE). Thermal interface material should be included at each interface (CONS1-INTERFACE) (Fig. 7).

**Fig. 7:**
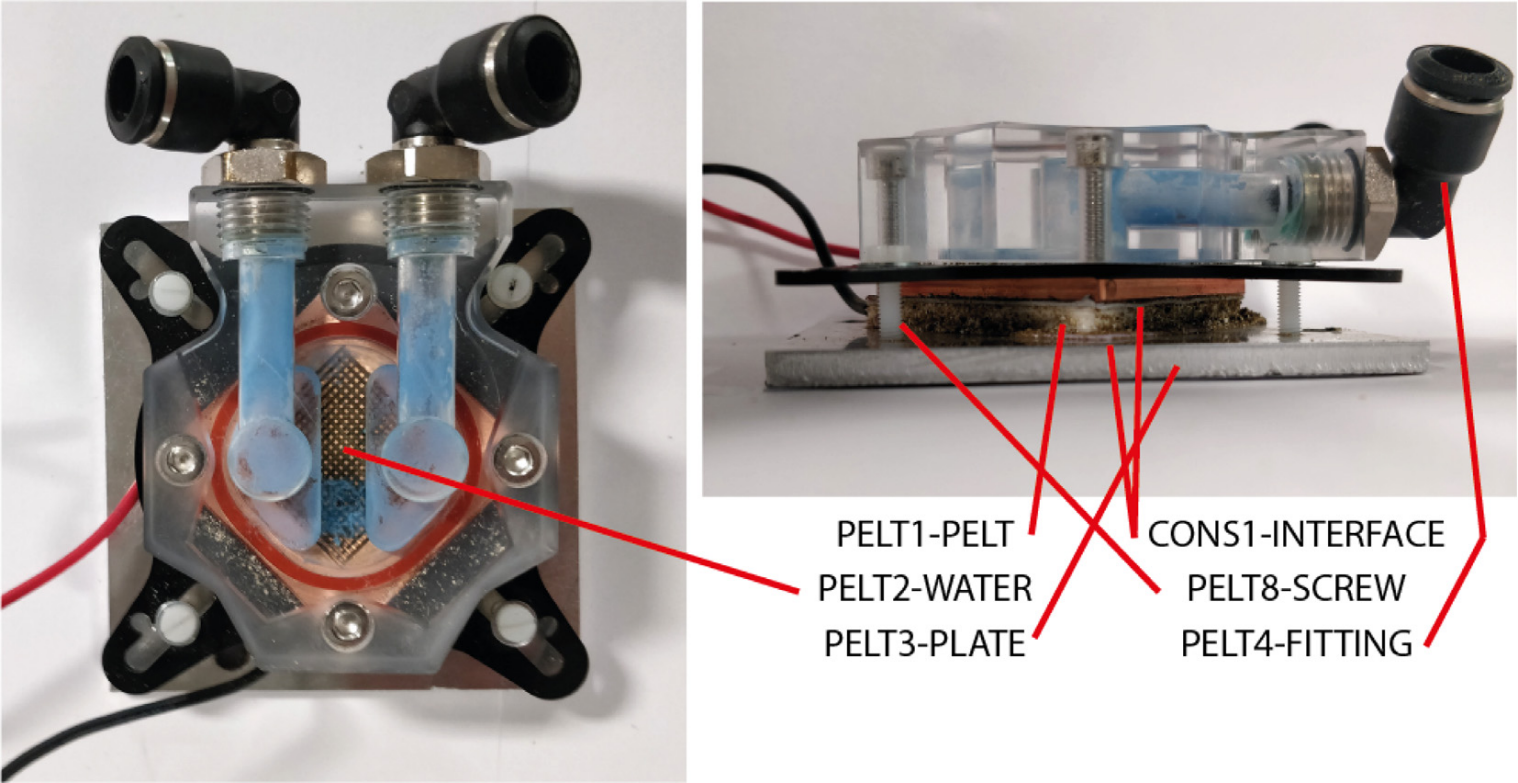
Peltier element assembly.
4. Carefully remove the cell from it’s packaging.
5. Cut a pieces of XPS foam (CONS2-FOAM) to raise the cell and support the tabs so that they can lie flat without stress or strain being imparted on the cell tabs and tab welds. Take into account the thickness of FIN3-TABBTTM under each tab. This sort of foam easily cuts with a hand saw or coping saw. The foam thicknesses and sizes required will depend on the cell being tested.
6. Clean the top surface of the bottom tab clamps (FIN3-TABBTTM) with solvent.
7. If using the fin separator (SETUP4-SEPARATOR and Section 5.4) ensure this is now attached to the fin-Peltier assemblies.

**Fig. 9:**
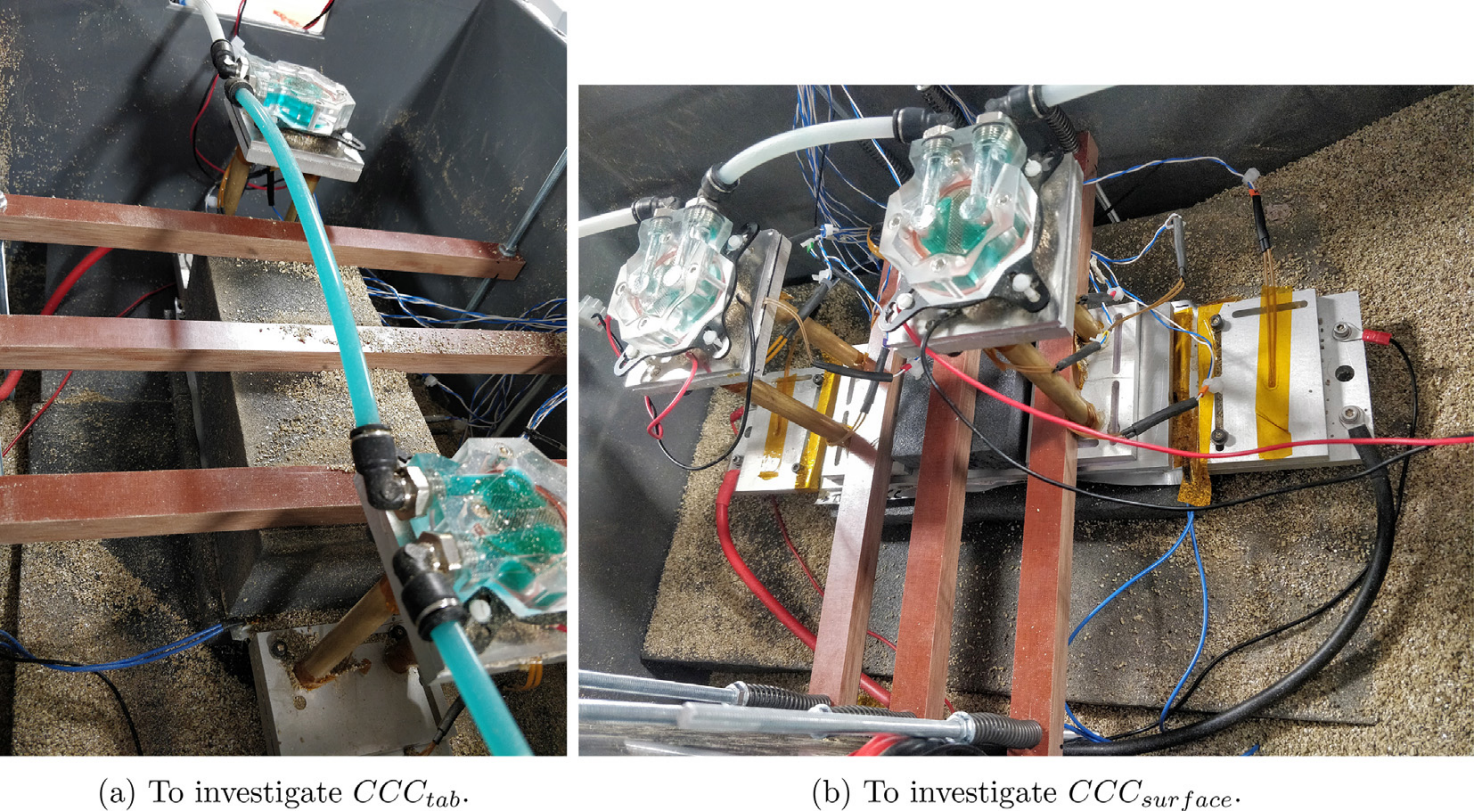
AMTE 20Ah example test setups.

If ${\mathrm{CCC}}_{\mathrm{tab}}$ is under investigation (Fig. 9a):

1. Place one GEN1-THERMO on the top and bottom surfaces of the cell at locations the furthest from each tab but at least 5 mm in from the outer edges of the electrode-stack. Secure with Kapton tape.
2. While ensuring negative tab remains insulated, remove the cover from the positive tab and clean the tab.
3. Using Kapton tap place 1× GEN1-THERMO at the tab centre. If separate temperature sensors are required for feedback to the Peltier controller place them either next to GEN1-THERMO or, if the tabs are small, elsewhere on the tab clamp surface.
4. Cut a piece of 1mm thick thermal interface material to the same size as the tab. To reduce pressure on the thermistor, remove the part of the interface under which the thermistor lies.
5. Clamp one fin assembly to the tab and corresponding (positive or negative) tab bottom plate.
6. Any areas of the fin set that could contact the other fin set during assembly should now be covered with insulating tape (Kapton or PVC) before the other tab is uncovered.
7. Repeat steps [2], [3], [4], [5] with the other tab.
8. Place the cell assembly in the box on supporting pieces of insulation.
9. Cut a piece of expanded polystyrene with thickness greater than 50mm, enough to cover the whole top surface of the cell. Place on top of the cell.
10. Depending on the size of the cell, one, two or three clamping bars will be required. Place the studding clamping rods through holes in the box such that bars are evenly spread across the insulation on top of the cell. Fit the clamping bars and springs to the rods as per Fig. 1. Use Eq. 2 to find the correct length to tighten the springs to exert the pressure suggested by the cell manufacturer. N.B. the springs given in this design can produce a maximum force of 169N in their linear range, i.e. 338N per bar.

If ${\mathrm{CCC}}_{\mathrm{surface}}$ is under investigation (Fig. 9b):

1. Place one GEN1-THERMO on the top and bottom surfaces of the cell, in the centre of the electrode-stack. Secure with Kapton tape.
2. While ensuring negative tab remains insulated, remove the cover from the positive tab and clean the tab.
3. Clamp the tab between the empty tab top plate (FIN4-TABTOP) and corresponding (positive or negative) tab bottom plate.
4. Any areas of the positive tab clamps that could contact the negative one should be covered in insulating tape (Kapton or PVC) before the other tab is uncovered.
5. Repeat steps [2], [3], [4] with the other tab.
6. Place the cell assembly in the box on supporting pieces of insulation.
7. Place an aluminium plate of thickness >8mm and just larger in width and length than the electrode-stack on top of the cell. Apply Kapton tape on any edges that might come into contact with tab clamps.
8. Place one or two fin/Peltier assemblies onto the aluminium plate with thermal interface material (CONS1-INTERFACE) or thermal paste across the whole contact.
9. Depending on the size of the cell and number of fin sets, one, two or three clamping bars will be required. Place the studding clamping rods through holes in the box such that bars are evenly spread across the insulation on top of the cell. Fit the clamping bars and springs to the rods as per Fig. 1. Use Eq. 2 to find the correct length to tighten the springs to exert the pressure suggested by the cell manufacturer. N.B. the springs given in this design can produce a maximum force of 169N in their linear range, i.e. 338N per bar.

**Fig. 10:**
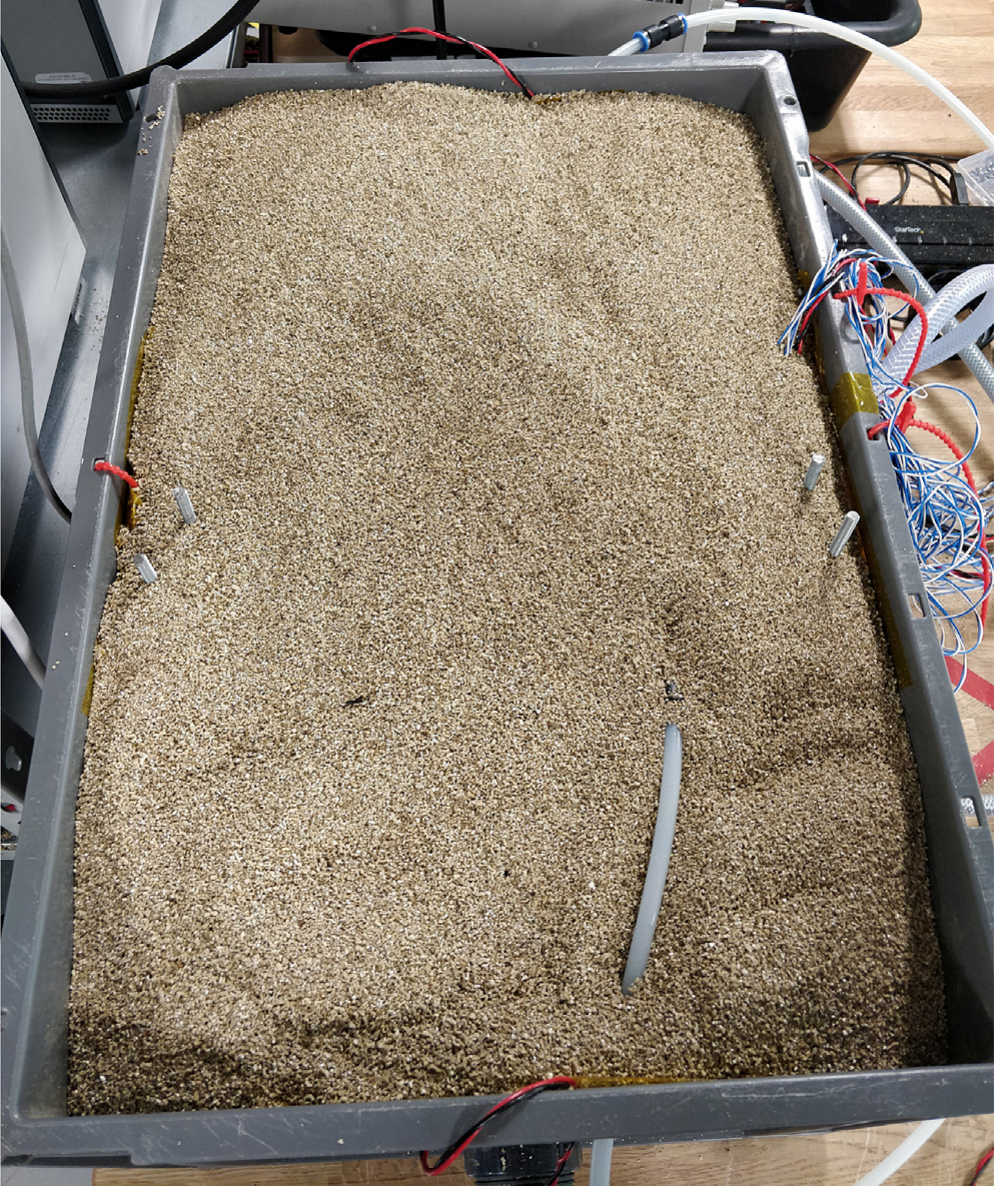
Test setup when ready to run.

For all experiments:

1. Cover any unused clamping rod holes in the box with tape.
2. Plug the water cooling blocks into the cooling system using PELT6-HOSE.
3. Plug the Peltier elements into the output on the Peltier control box that correspond to the thermistor directly under that Peltier.
4. Measure the contact resistance between the cables and the cell tabs, $R_{\mathrm{contactPos}}$ and $R_{\mathrm{contactNeg}}$. This can be done by using the battery cycler to pass a 10A current through the cell and measuring the voltage drop with a precision voltmeter. Contact between the probe and the tab can be made through the slots in the cell tab clamps.
5. Fill the box with vermiculite and gently agitate the box to allow it to settle (Fig. 10).

(2) $$L=L_{F=0}-\frac{F}{k}=85.5-\frac{F}{3.61}$$

### Testing procedure

For measuring the ${\mathrm{CCC}}_{\mathrm{tab}}$, the tab temperature, $T_{\mathrm{set}}$ should be kept close to ambient temperature. This reduces heat losses through the cables, an area we have identified as a source of error for this measurement.

For measuring the ${\mathrm{CCC}}_{\mathrm{surface}}$, the cell top temperature, $T_{\mathrm{set}}$, should be kept $1^{\circ }C$ below ambient temperature. This reduces heat losses through the bottom of the cell, an area we have identified as a source of error for this measurement.

To run the experiment:

1. On the battery cycler prepare the test protocol given in Fig. 11. See note below about selection of appropriate currents.

**Fig. 11:**
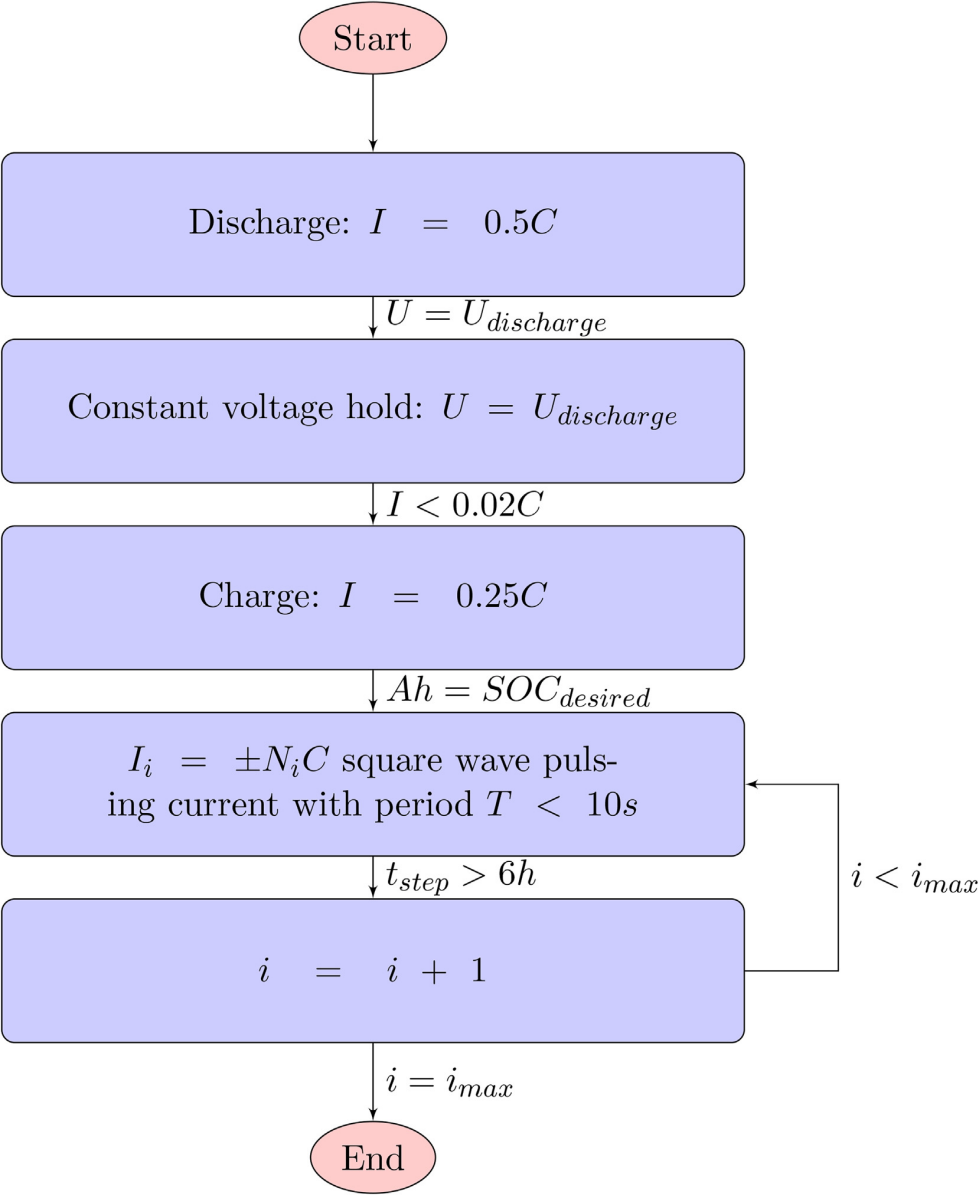
Battery test protocol. $N_{i}$ is given in Eq. 4.
2. Turn on the closed circuit water cooling.
3. Turn on the Peltier temperature control.
4. Begin temperature recording.
5. Begin the test protocol.

For the test measurements to be accurate the cell must be tested at pulsing currents such that the range of heat generation in the cell spans the full range of measurable heat rates in the chosen fin sets. In order to minimise error it is also recommended that temperature gradient across the cell be greater than $0.5^{\circ }C$. Precise prediction of heat generation using cell datasheets is not always possible so an iterative approach may need to be taken. One method is to perform the test as normal but using only a 1C pulsing current. Record the heat generation, $Q_{1C}$, and temperature gradient, $\Delta T_{1C}$, at 1C. Then use Eq. 3 to find the minimum and maximum C rates, *N*, that should be used. In cases where small cell temperature gradients are the limiting factor the same analysis can be performed but replacing $Q_{1C}$ with $\Delta T_{1C}$ and $Q_{\min}$ with $\Delta T_{\min}$ to find $N_{\min}$.

(3) $$\begin{array}{cc} R & =Q_{1C}/1\\ N_{\min} & =\sqrt{Q_{\min}/R}\\ N_{\max} & =\sqrt{Q_{\max}/R} \end{array}$$

It is recommended that a square root progression in current magnitude is used so that there is a linear progression in heat generation, since heat generation is expected to increase with the square of current magnitude. An example to give 6 C-rates in the range is given in Eq. 4.

(4) $$I_{i}=N_{i}C\;\mathrm{where}\;N_{i}=\{\sqrt{N_{\min}^{2}},\sqrt{N_{\min}^{2}+1\frac{N_{\max}^{2}-N_{\min}^{2}}{5}},\sqrt{N_{\min}^{2}+2\frac{N_{\max}^{2}-N_{\min}^{2}}{5}},\ldots ,\sqrt{N_{\max}^{2}}\}$$

### Cell removal

1. Check battery cycling has stopped.
2. Shut down Peltier control.
3. Shut down closed circuit water cooling loop and disconnect hoses.
4. Unscrew the vermiculite drain and pour out the vermiculite.
5. Loosen clamping springs and remove clamping bars and anything on top of the cell.
6. Undo tab clamps one at a time taking care to cover tabs with insulating tape as soon as they are exposed.
7. Remove cell.

### Data processing

1. Take the time average temperature readings for the last 10 min (600s) at each pulsing current (example for surface cooling CCC temperature data shown in Fig. 12).

**Fig. 12:**
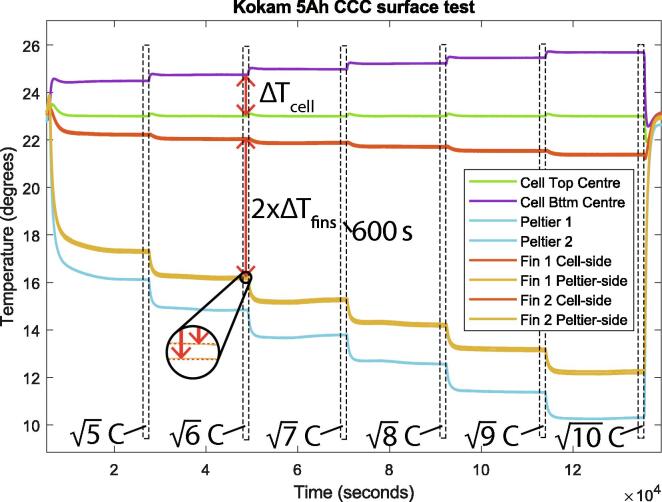
Kokam 5Ah data for surface cooled test. Cell top surface is held at a constant $23^{\circ }C$. The test was performed with 2x single 1.25W–2.5W fin sets.
2. For each fin, $\Delta T_{\mathrm{fin}}$ should be found. Each $Q_{\mathrm{fin}}$ can then be calculated by Eq. 1. This must be done for each test conducted, i.e. for each current magnitude. See example in Fig. 12, where each region for data extraction is annotated.

For a surface cooled test:

1. Calculate the $\Delta T_{\mathrm{cell}}$ by comparing the time averaged temperatures. Where multiple locations have been measured take the average $\Delta T_{\mathrm{cell}}$.
2. Sum every $Q_{\mathrm{fin}}$ in the system to give $Q_{\mathrm{tot}}$.

For a tab cooled test:

1. Take the average of the cell top and bottom temperature at the point furthest from the tabs to give $T_{\mathrm{cell}}$
2. Find the temperature difference between each tab and $T_{\mathrm{cell}}$ to give $\Delta T_{\mathrm{pos}}$ and $\Delta T_{\mathrm{neg}}$.
3. Calculate $\Delta T_{\mathrm{cell}}$ as the average of $\Delta T_{\mathrm{pos}}$ and $\Delta T_{\mathrm{neg}}$.
4. Take the total heat output from each tab (calculated in step 2), $Q_{\mathrm{pos}}$ and $Q_{\mathrm{neg}}$. Deduct from each the resistive heat, $Q_{\mathrm{ohmic}}$, generated at each tab under each pulsing current using the tab contact resistance found during setup and the formula $Q_{\mathrm{ohmic}}=I^{2}R$. This gives a new $Q_{\mathrm{posNet}}$ and $Q_{\mathrm{negNet}}$ to be used in the next step that only includes heat generated in the cell (Eq. 5).
5. Sum $Q_{\mathrm{posNet}}$ and $Q_{\mathrm{negNet}}$ to give $Q_{\mathrm{tot}}$

(5) $$\begin{array}{cc} Q_{\mathrm{ohmicPos}} & =I^{2}R_{\mathrm{contactPos}}\\ Q_{\mathrm{ohmicNeg}} & =I^{2}R_{\mathrm{contactNeg}}\\ Q_{\mathrm{posNet}} & =Q_{\mathrm{pos}}-Q_{\mathrm{ohmicPos}}\\ Q_{\mathrm{negNet}} & =Q_{\mathrm{neg}}-Q_{\mathrm{ohmicNeg}} \end{array}$$

**Fig. 13:**
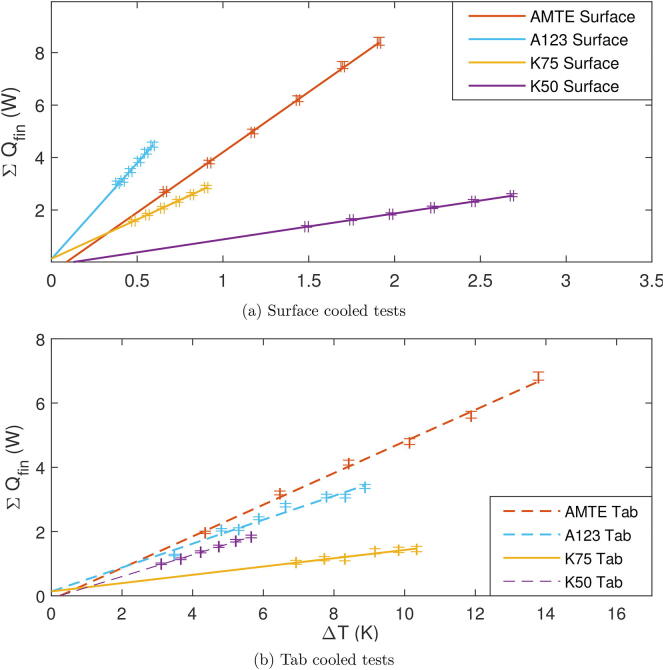
Q vs T for all CCC tests.

In either case plot $\Delta T_{\mathrm{cell}}$ against $Q_{\mathrm{tot}}$. The gradient of the line of best fit through the points gives the *CCC*. An example of this is shown in Fig. 13, which is discussed in the following section.

## Validation and characterisation

Validation of results was performed by comparison with values collected using the systems described in [5] and [6]. The cells tested are given Table 1, and their assigned references will be used to refer to them in this analysis (Table 2).

**Table 1:** Cells tested as part of system validation.

Ref.	Manufacturer	Capacity	Product number	Electode-stack dimensions	Tab dimensions
AMTE	AMTE	20Ah	ULTRA POWER	(L) 252 mm x (W) 97 mm x (t) 6.9 mm	(L) 40 mm x (W) 60 mm x (t) 0.5 mm
A123	A123	19.6Ah	AMP20M1HD-A	(L) 227 x (W) 160 mm x (t) 7.3 mm	(L) 30 mm x (W) 35 mm x (t) 0.4 mm
K75	Kokam	7.5Ah	SLPB75106100	(L) 102 mm x (W) 107 mm x (t) 7.9 mm	(L) 13 mm x (W) 5 mm x (t) 0.3 mm
K50	Kokam	5Ah	SLPB11543140H5	(L) 143 mm x (W) 43 mm x (t) 11.7 mm	(L) 20 mm x (W) 20 mm x (t) 0.3 mm

**Table 2:** Cells CCC values

Ref.	New tab CCC (W/K)	New surface CCC (W/K)	Previous tab CCC (W/K)	Previous surface CCC (W/K)
AMTE	0.48	4.59	0.45	3.54
A123	0.37	7.33	0.26	4.70
K75	0.13	2.99	n/a	1.91
K50	0.34	1.00	0.33	1.00

Fig. 13a shows all results from ${\mathrm{CCC}}_{\mathrm{surface}}$ tests conducted using the presented apparatus. Fig. 13b shows all results from the ${\mathrm{CCC}}_{\mathrm{tab}}$ tests. Table 1 provides results. As previously introduced, the CCC values are simply the gradient of each line shown in Fig. 13a and b respectively.

It is important to note that it is not the intention of this analysis to evaluate the presented apparatus’s performance against the system for measuring the ${\mathrm{CCC}}_{\mathrm{surface}}$ that was used in [6]. The former system had known errors which are discussed extensively in the original paper and may be summarised as:

1. Heat loss through the ‘hot’ surface of the cell is unavoidable because the insulation is not perfect. Whilst measures to account for this heat loss are implemented, they do not entirely eliminate the source of error. Proposals were made to improve the thermal control system in order to ensure the cell ‘hot’ surface may be held at ambient temperature, thus eliminating the temperature gradient to the ambient, and thus any losses from the ‘hot’ surface to the ambient.
2. Temperature difference across the cell under test is small and therefore oscillations in temperature at steady state, due to the substandard performance of the Peltier control system, lead to significant imprecision in extracting the true temperature difference.
3. Imprecise methods of compressing the cooling fins onto the cell leads to deviating thermal interface performance (from cell ‘cooled’ surface to fins), and thus deviating thermal performance of the entire apparatus from one test to the next.

The presented apparatus has addressed the previous limitations through improved design and procedural methods:

1. Better insulation (vermiculite fills the complex topology of the apparatus) to reduce heat loss.
2. Improved choice of thermal control locations to ensure there is not a significant temperature gradient from the ‘hot’ surface of the cell to the ambient.
3. Compression bars with controllable force to ensure the desired pressure is always exerted on the cell, from the cooling plate and fins.

As a result, the measured values of ${\mathrm{CCC}}_{\mathrm{surface}}$ were expected to deviate significantly from the past measurements, and the new values are now viewed as a more reliable measure of each cell’s thermal performance.

Strength to this argument may be found by considering the ${\mathrm{CCC}}_{\mathrm{surface}}$ measurements from K50, which do not see significant deviation from the previous setup (1.00 W/K) to the current (1.00 W/K). This cell is significantly smaller than the other three cells tested, and subsequently error due to losses through the ‘hot’ surface of the cell was expected have a less dominant effect. Following this trend, the largest cell (A123) sees the greatest change, the previous measured value (4.70 W/K) is 36% smaller than the new value (7.33 W/K)

The methods presented in [5] are significantly more robust, and as a result the apparatus set out in this paper to measure the ${\mathrm{CCC}}_{\mathrm{tab}}$ is conceptually very similar that used previously. There is very good agreement with the previous and current sets of results for the AMTE cell and the K50 cell, with deviation from previous to current at 6% and 3% respectively. These cells have large tabs (with respect to their electrode-stack size) located at either end of the cell, and therefore are ideally designed to limit the temperature gradient along the electrode-stack length. This means that it is easy to maintain a small temperature difference from the cell surface to the ambient, and thus losses to the ambient are small. All this creates a good scenario for a robust CCC experiment to be conducted.

The larger deviation observed for the A123 cell (30%) represents the inadequacy of this cell to be tab cooled, and thus the difficulty in measuring a consist and sensible value for the tab CCC. The poor cell design is discussed at length in [2]. The A123 has small tabs, located very close to one another, at one end of a large electrode-stack. This leads to a large temperature gradient along the length of the cell. As a result, it is impossible to maintain a negligible temperature difference from all external surfaces of the cell to the ambient, since the temperature of the external surfaces varies so much across the cell’s geometry. Therefore, losses to the ambient are inevitable, and add an unknown error to the CCC measurement process. Finally, it must be mentioned that the tabs on K75 were so small that an experiment could not be conducted within the scope of this project. K75 is not designed to be tab cooled, and as such this is not seen as a limitation to the presented apparatus.

## Declaration of Competing Interest

The authors declare that they have no known competing financial interests or personal relationships that could have appeared to influence the work reported in this paper.

## References

[1] Nazari A., Farhad S. (2017). Heat generation in lithium-ion batteries with different nominal capacities and chemistries. Appl. Therm. Eng..

[2] O. Dondelewski, et al., The role of cell geometry when selecting tab or surface cooling to minimise cell degradation, eTransportation 5 (2020).

[3] Fleischhammer M., Waldmann T., Bisle G., Hogg B., Wohlfahrt-Mehrens M. (2015). Interaction of cyclic ageing at high-rate and low temperatures and safety in lithium-ion batteries. J. Power Sources.

[4] Offer G., Patel Y., Hales A., Bravo Diaz L., Marzook M.W. (2020). Cool metric for lithium-ion batteries could spur progress. Nature.

[5] Hales A. (2019). The Cell Cooling Coefficient: A Standard to Define Heat Rejection from Lithium-Ion Batteries. J. Electrochem. Soc..

[6] Hales A., Marzook M.W., Bravo Diaz L., Patel Y., Offer G. (2020). The Surface Cell Cooling Coefficient: A Standard to Define Heat Rejection from Lithium Ion Battery Pouch Cells. J. Electrochem. Soc..

[7] Hales A. (2020). The Cell Cooling Coefficient as a design tool to optimise thermal management of lithium-ion cells in battery packs. eTransportation.

[8] Dupré minerals, Dupré Exfoliated Vermiculite – Datasheet, 2019. Available at:https://www.dupreminerals.com/wp-content/uploads/2017/04/vermiculite-datasheet.pdf. Accessed 18/06/2021.

[9] G. White, F. Russell, R. Prosser, G. Offer, Y. Patel, OpenPATs: An open-source low-cost Peltier element advanced temperature control system PAPER IN PREPARATION.

[10] Lakatos Á., Kalmár F. (2013). Investigation of thickness and density dependence of thermal conductivity of expanded polystyrene insulation materials. Mater. Struct..

